# Recycling and sustainable applications of waste printed circuit board in concrete application and validation using response surface methodology

**DOI:** 10.1038/s41598-023-43919-9

**Published:** 2023-10-02

**Authors:** M. Vishnu Priyan, R. Annadurai, Kennedy C. Onyelowe, George Uwadiegwu Alaneme, Nimay Chandra Giri

**Affiliations:** 1 Department of Civil Engineering, SRM Institute of Science and Technology, Kattankulathur, Chengalpattu, 603203 Tamil Nadu India; 2 Department of Civil Engineering, Michael Okpara University of Agriculture, Umudike, Nigeria; 3 Department of Civil, School of Engineering and Applied Sciences, Kampala International University, Kampala, Uganda; 4 Department of Electronics and Communication Engineering, Centurion University of Technology and Management, R. Sitapur, 752050 Odisha India

**Keywords:** Engineering, Materials science

## Abstract

The present investigation aims to examine the mechanical and durability properties of concrete that has been reinforced with a waste printed circuit board (WPCB) towards a low-carbon built environment. It assessed the fresh and hardened characteristics of the low-carbon concrete reinforced with WPCB fibres, after a curing period of 7 and 28 days. The evaluation was done by quantifying slump, compressive strength, split tensile strength, flexural strength, sorptivity, rapid, and acid tests. It further analysed eleven discrete concrete mixes with WPCB fibres at a weight percentage ranging from 1 to 5% in the cement mixture. The results indicate that incorporating WPCB fibre into concrete improves its mechanical strength. The results revealed that incorporating 5% WPCB fibre yielded the most favourable outcomes. The properties of WPCB fibre-reinforced concrete have been theoretically validated through Response Surface Methodology (RSM), which employs various statistical and mathematical tools to analyse the experimental data. The results derived from RSM were compared with the experimental results. It was found that the RSM model demonstrated a high level of accuracy (R^2^ ≥ 0.98) in validating the mechanical properties of WPCB fibre concrete. The statistical model exhibited no indication of prediction bias and demonstrated a statistically significant outcome, with a p-value below 0.5.

## Introduction

In contemporary times, the issue of sustainability has emerged as a significant preoccupation for humanity, especially in the Eco-Friendly Built Environment (EFBE) in line with the requirements of the Conference of the Parties (COP27) and United Nations Sustainable Development Group (UNSDGs). Based on this premise, global resources are steadily diminishing due to housing demands while the human populace is experiencing a rapid surge. The efficient utilization of resources holds significant importance in this regard^1–5^. There have been many attempts to reuse waste products creating environmental hazards such as fibre and filler material in the construction industry. A brief literature Review indicates that studies have attempted to incorporate materials like rubber tree seed cells, textile-based carbon nanotube composites, waste marble powder, coconut fibre, recycled steel wire waste tyres, steel fibres, etc. There also has attempts to utilise material like used engine oil and thermoset plastic to develop sustainable concrete^6–10^. These studies have proved that enhancement in the performance characteristics can be achieved by reusing these waste materials providing an option for sustainable development through green products^11–17^. Massively, electronic waste (e-waste) is generated as millions of electronic gadgets are rendered obsolete yearly^18^. United Nations Environment Programme (UNEP) predicts that the quantum of e-waste generated in India could increase by 500% over the next decade. The global production of Waste Electrical Equipment (WEE) is rising owing to the rapid technological obsolescence of electronic products and the availability of newer products at reasonable prices. WEE contains numerous economically valuable and environmentally hazardous metals and synthetic compounds, but it also is composed of toxic substances that pose a significant hazard to the environment and society. Developed nations have incorporated reuse laws and regulations into their waste electrical and electronic management policies while developing countries lag^19^. In emerging economies, households discard WEE through improper disposal, leading to environmental pollution and health risks^20^. The waste materials are often discarded in nearby fields^21,22^. Mishandling e-waste leads to harmful composites contaminating the local environment, including soils, sediments, dust and plants^23^. Critical solid waste is also disposed of following improper disposal techniques^24^. The electronic waste generated from electrical and electronic devices industries and households consists of Liquid Crystal Display (LCD) screens (11.9%), Personal Computers (PCs) (18.8%), mobile phones (21.3%) and Cathode Ray Tube (CRT) Televisions (7%). Only 10% of electronic waste is recycled. This waste can be recycled using various technologies or disposed of directly in a landfill or an incinerator^25^. E-waste can be recycled or reused, but improper techniques and equipment for disposal may harm the environment and humans. Reuse is repurposing a product for a purpose other than its original design. Reusing can be done using various product life-extension strategies, such as repair, refurbishment and remanufacturing. Reusing e-waste can also extend the product's lifespan.

Printed circuit boards (PCBs) provide electrical interconnections between elements and report for approximately 4% of all electronic waste^26,27^. Waste Printed Circuit Boards (WPCBs) are an extremely heterogeneous product category in size, material composition and shape^28^. WPCBs contain many carbon and toxic substances, including heavy metals and brominated flame retardants, which are harmful to the environment if not properly treated. Constant technological advances have hastened the regular replacement of electrical and electronic devices, significantly increasing the quantity of discarded WPCBs^29^. In the recent past, WPCB manufacturing reached a global growth rate of 8.7%, while in Southeast Asia and China, WPCB manufacturing grew at 10.8% and 14.4%, respectively^30^ China currently produces 40% of the world's WPCBs. WPCBs contain many metals, including carbon, copper, aluminium, iron, tin and lead, and non-metals, such as thermosetting resins and glass fibres^29–31^. Metal extraction is done as part of WPCB recycling. Hydrometallurgical, pyrometallurgical and mechanical methods are used to recover metals from waste PCBs. The metals in waste PCBs are recycled/recovered using mature processes like Hydrometallurgical, pyrometallurgical and mechanical methods for industrial and commercial purposes as secondary raw material. Hydrometallurgical is a method which utilises various acid to recover metals from waste PCBs and has recovery efficiency of 60–70%^32–34^. Pyrometallurgical is a process using high temperatures to recover metals like gold, silver and copper etc. from waste PCBs and is has recovery efficiency of 85–95%^35–37^. Although the above-mentioned process and not environmentally friendly. Hydrometallurgical has the disposal problem^38–40^ and pyrometer is considered as an energy-intensive process^41,42^. The mechanical methods generate residue of waste PCB in various sizes, further worsening solid waste management. The above-mentioned are only concentrating on the recovery of metals present in the Waste PCB, the board which contains fibre which further increases the load on environmental pollution and intensifies the problem in solid waste management. However, reusing the substantial amounts of non-metals that comprise approximately 70% of WPCBs presents a formidable challenge^43^. Currently, non-metals reprocessed from waste WPCBs are generally disposed of in incinerators or landfills, which leads to hazardous environmental effects^44–46^. Reusing non-metals from WPCBs has become a significant challenge for e-waste management and a source of worry in environmental protection and resource recycling^47–49^. Yokoyama and Iji^50^ investigated the recycling of non-metals as fillers in resin-type building materials. Mechanical and Vicat Softening Temperature (VST) tests were employed to recycle non-metals used as reinforcing fillers in thermoplastic resin matrix composites^51^. Polypropylene (PP) composites show a maximum increase of tensile, tensile strength, flexural strength and flexural modulus by 62.9%, 28.4%, 87.8% and 133.0%, respectively. Without violating environmental regulations, 30% of non-metals recycled from WPCBs can be included in polypropylene composites. Hong and su^52^ utilized non-metallic fillers as reinforcements in polyester composites. Incorporating WPCBs altered the Unsaturated Polyester (UP) resin's free radical reaction and reduced the initial curing. Because of the energy dissipation effects of epoxy resins (rigid) and glass fibres in the WPCB, WPCB-modified UP resins had a high glass transition temperature (*T*_g_). They were stronger and tougher than plain UP. Franz^53^ stated that using non-metals as a thermoplastic material would be an ideal recycling solution. The strategy involved reusing non-metals to create thermoplastic housing. Unfortunately, this approach runs counter to current product miniaturisation trends.

WCPB wastes are non-biodegradable materials and could persist on the earth for hundreds or even thousands of years after they are discarded. Reusing these materials in the building and construction industry reduces the demand for raw materials extracted from natural sources^54–56^. Using waste materials in construction also prevents environmental degradation caused by their disposal and affirms sustainable construction practices^57–62^. The effective management of various waste production processes has come under increased scrutiny in recent years to make the building industry environmentally sustainable. One of the critical considerations of waste management strategies is using waste materials instead of natural materials as raw materials^63^. The primary benefits of recycling are decreasing the pollution released into the environment, reducing the amount of trash discarded and preserving natural resources^25,63–67^. Over the past few years, the response surface method (RSM) has been modified to solve various issues in civil engineering, such as forecasting different characteristics, evaluating concrete structures' capacity to support loads, modelling material behaviour, optimising and controlling structures and monitoring groundwater^68^. RSM is a statistical and mathematical tool set. It can model and evaluate practical problems^69^. Despite its everyday use in trial and optimization design, this tool set has seen restricted application in the concrete industry^70^. As per the literature reviewed by the investigators, research on WPCB fibre-reinforced concrete was focused primarily on using WPCB as a substitute for natural aggregates and cement^54–56,61,62,66,67,71–73^.

The viability of using WPCBs in concrete as fibre strips cut from WPCBs was investigated For Aspect Ratio (AR) 10 and 20 to enhance the mechanical properties^74^. The problem stated in support of this project is that the increased generation, management, and disposal of E-waste (WPCBs) causes various environmental and health issues. This study aims to use WPCBs as fibre as an alternative to conventional fibres, potentially lowering construction costs and demand for natural resources and reducing the environmental hazard to address and sustainably prevent the abovementioned problems. This investigation experimentally examines the durability characteristics and structural strength aspects of WPCB fibre-reinforced concrete of AR30 and AR40. It validates the experimental results by comparing them with the theoretical values for mechanical parameters of WPCB fibre-reinforced concrete obtained using the Response Surface Methodology.

## Experimental program

### Materials

**Figure 1:**
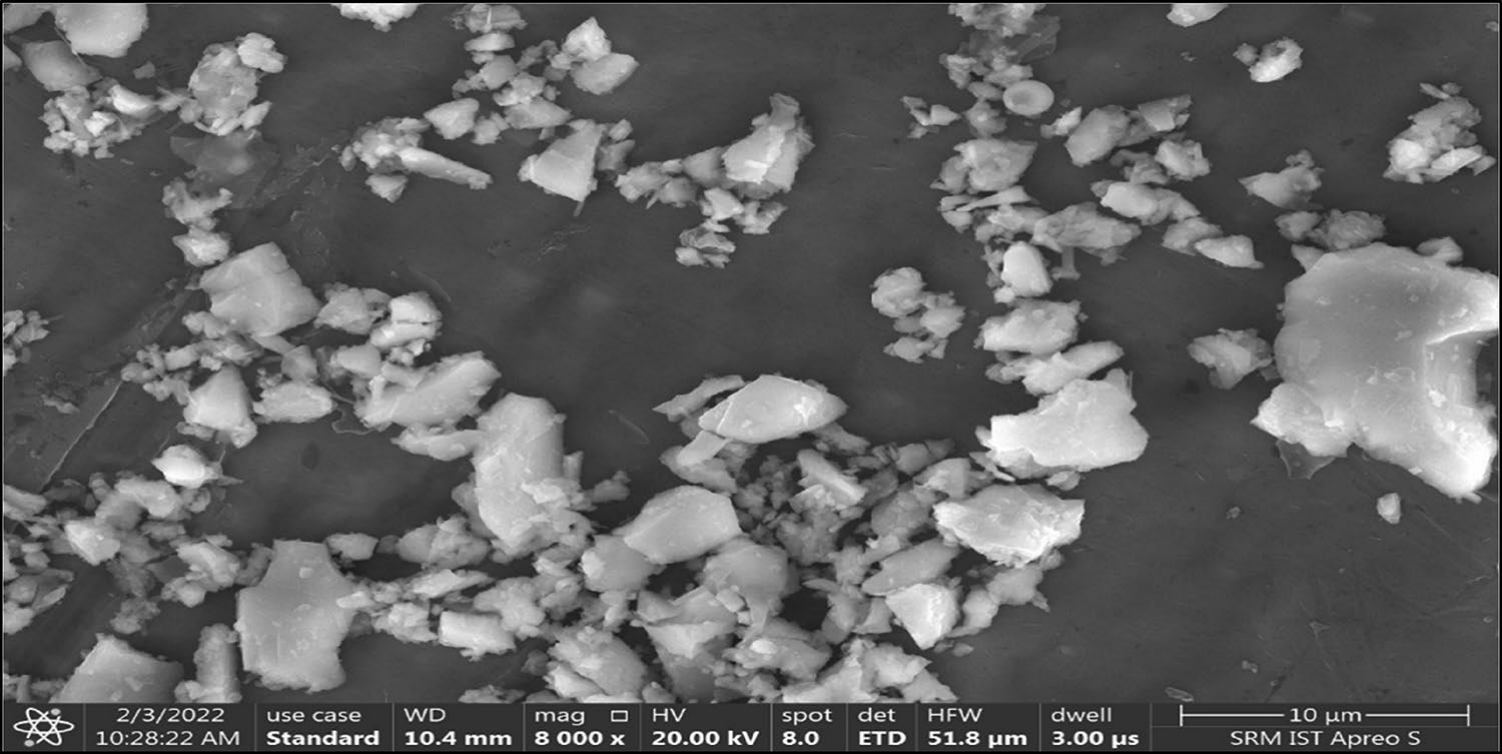
SEM image of OPC 53-grade cement.

The 53-grade Ordinary Portland Cement (OPC) utilised in this research was procured locally and conformed to BIS 12269-2013 standard^75^. Calcium oxide (CaO) was the main composition with a specific gravity of 3.14. Table 1 summarises some of OPC's physical and chemical properties. OPC 53 grade cement has a particle size distribution with a mean particle size of 0.0242 mm. The scanning electron microscope (SEM) image of OPC is shown in Fig. 1. The cement particle has an angular shape and a rough surface. This study utilized M-sand from a local supplier with a 4.75 mm maximum particle size, a specific gravity of 2.58, a fineness modulus of 2.98, and a bulk density of 1672 kg/m^3^. Coarse aggregate with a maximum particle size of 10 mm, a specific gravity of 2.72, fineness modulus of 7.11 and bulk density of 1548 kg/m^3^. All aggregates conformed to BIS 383-2016 standard^76^ Conplast SP430, a commercially available superplasticizer additive containing sulfonated naphthalene polymers, was used to achieve the desired workability that met the BIS 9103-1999 standards^74,77^. In addition, tap water was used to mix and cure the concrete.

**Table 1:** OPC's chemical composition and physical characteristics.

Chemical composition	Mass (%)
Calcium oxide (CaO)	64.23
Ferric oxide (Fe_2_O_3_)	3.05
Sodium oxide (Na_2_O)	0.15
Particle size D_v,50_ (μm)	24.1
Potassium (K_2_O)	0.36
Silicon dioxide (SiO_2_)	19.34
Magnesium oxide (MgO)	1.53
Aluminium oxide (Al_2_O_3_)	4.76
Sulphur trioxide (SO_3_)	2.01
Other properties	
Loss on ignition	1.65
Density (kg/m^3^)	1344
Specific gravity	3.14
Specific surface area (cm^2^/g)	2039

### Printed circuit board fibre

**Figure 2:**
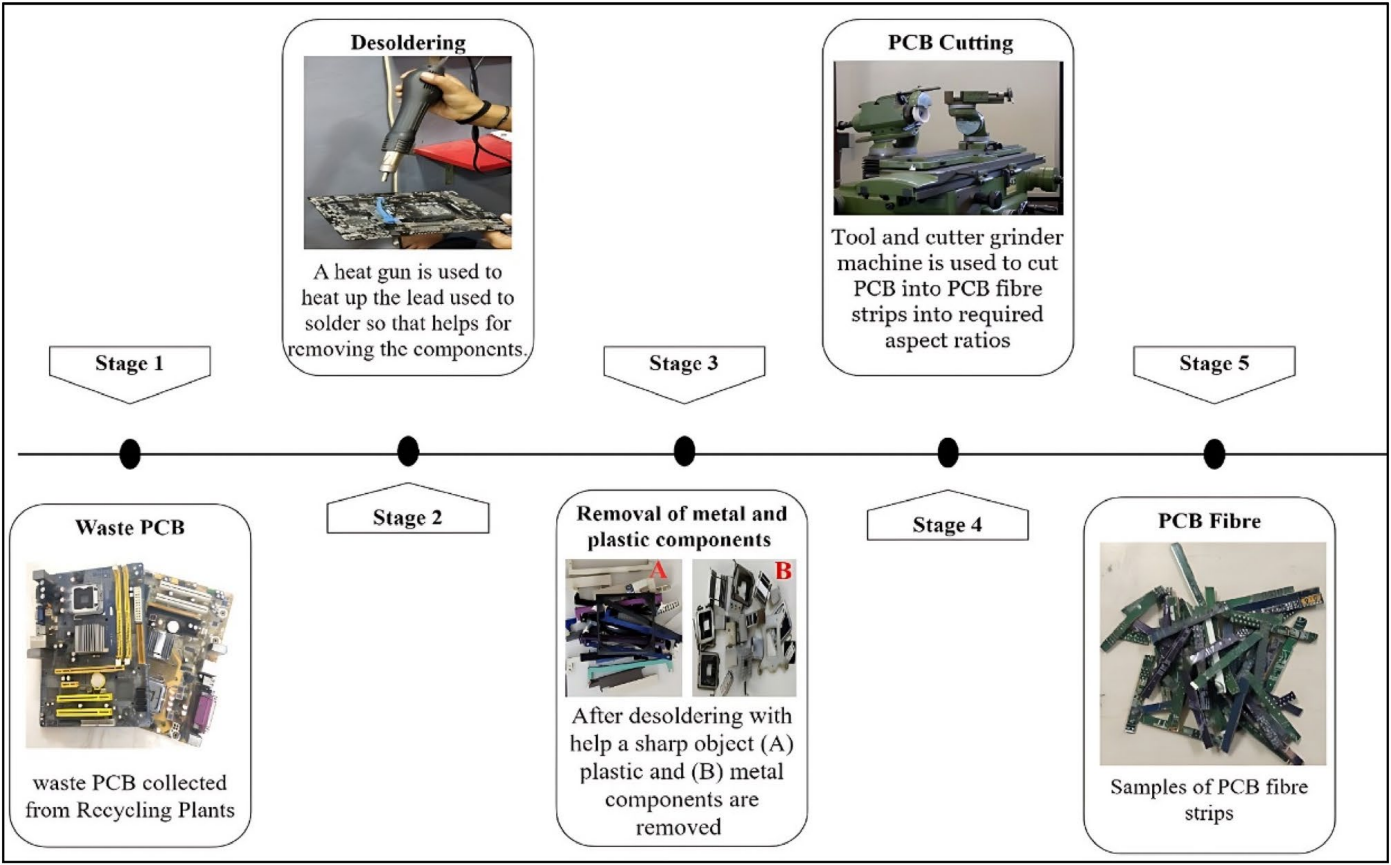
Process of making WPCB fibre.

The FR4-type WPCB used in this study was sourced from a local recycling facility and obtained from old personal computers. It is essential to clean discarded Printed Circuit Boards (PCBs) to ensure their safe and effective disposal or reuse^78^. After cleaning, external components were extracted through desoldering using a heat gun. Once chips, diodes, and other metallic components, including mechanical disassembly and removal of components that can be reused or recycled, were removed, the unadorned waste PCB was transformed into fibers with dimensions specified in Table 2 using various tools and a grinding machine. The processing steps of making WPCB is shown in Fig. 2.

**Table 2:** Physical properties and dimension of WPCB fibres.

Physical properties	Dimension
Length of WPCB fibre (mm)	40 and 45
Width of WPCB fibre (mm)	1.5 and 1
The thickness of WPCB fibre (mm)	1.6
Aspect ratio (L/W)	30 and 40

**Figure 3:**
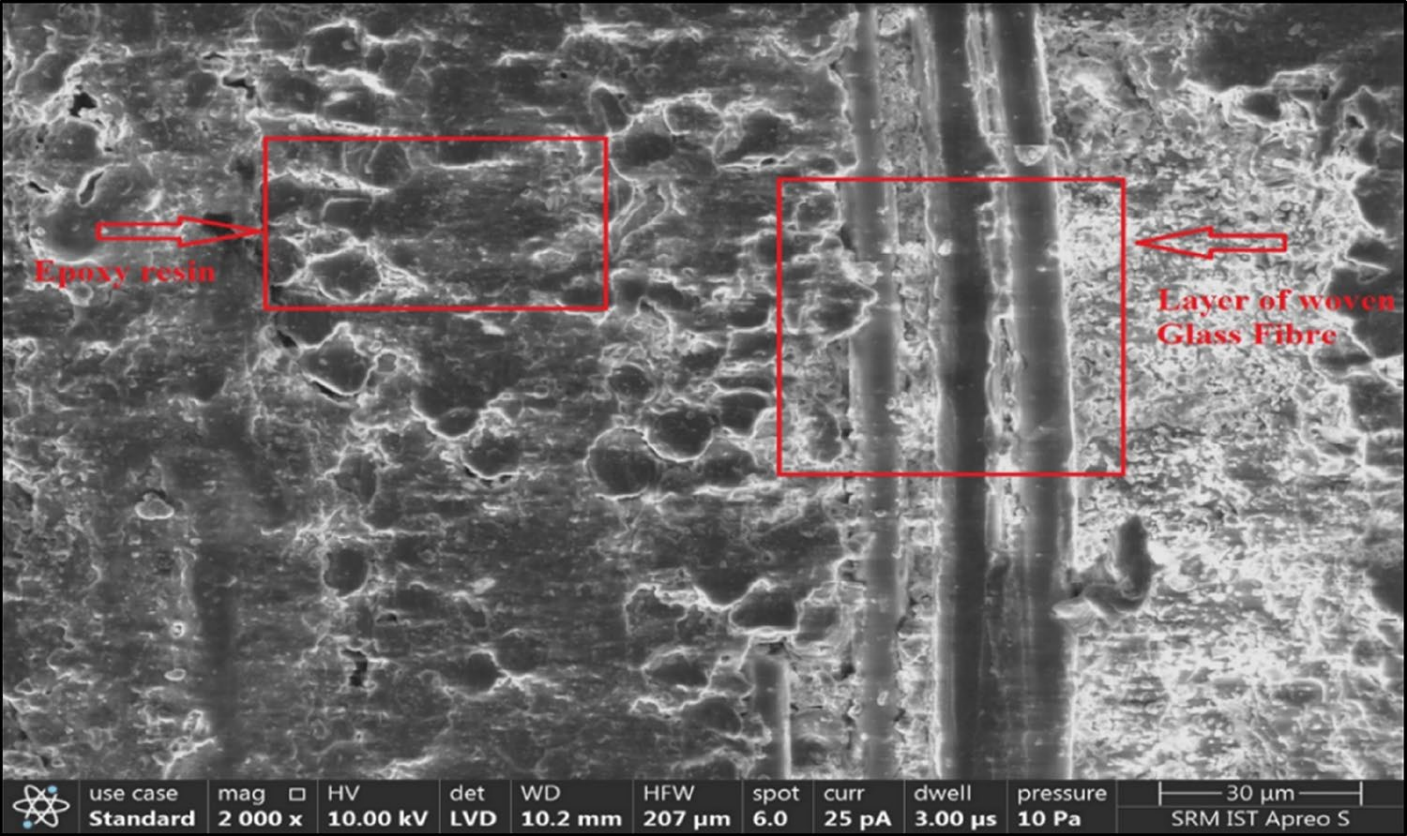
SEM image of WPCB fibre.

**Figure 4:**
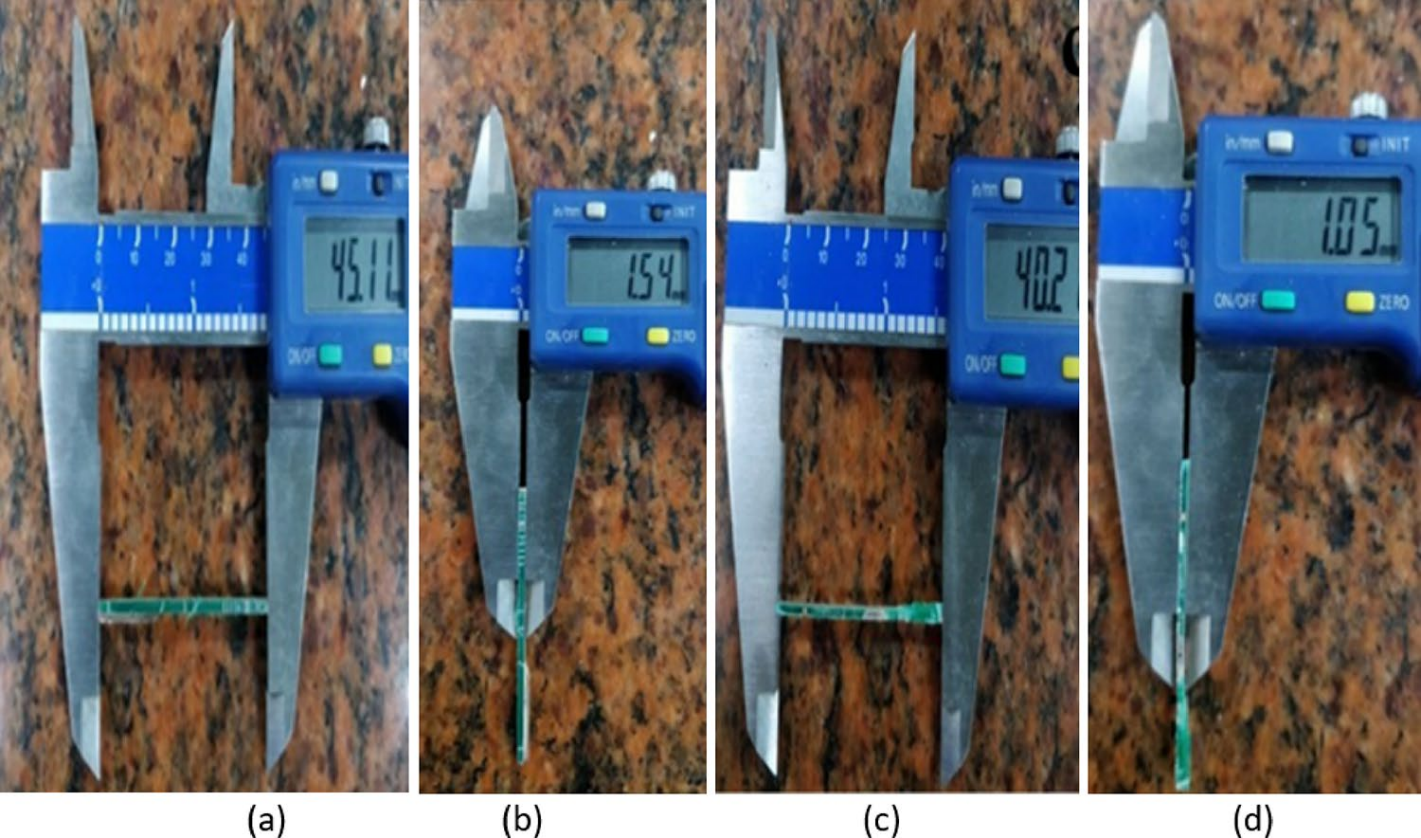
Length and width WPCB fibres: AR30 (**a**) and (**b**), AR40 (**c**) and (**d**).

The main composition of the WPCB is silica, and its specific gravity is 2.68. The physical properties and dimensions of WPCB fibre are shown in Table 2. An SEM image of the WPCB fibre is illustrated in Fig. 3. The image shows that WPCB fibre has layers of woven glass fibre bound to epoxy resin. The WPCB fibres have two aspect ratios, AR30 and AR40, used in the study (Fig. 4). These dimensions were determined based on previous research^74,79–81^.

### Mix proportion and procedures

In this study, the WPCB fibre concrete mix proportion was divided into three different mixes based on the percentage of added fibre. The control variables included cement, fine aggregates, coarse aggregates and three different weights of superplasticizers (550, 708 and 813 kg/m^3^). Two aspect ratios of the WPCB fibre (AR30 and AR40) were tested; therefore, ten unique mixtures were prepared by adding varying amounts of WPCB fibre to the weight of cement (1%, 2%, 3%, 4%, and 5%). In all these 11 different mixes, the proportion of the superplasticizer was fixed as 1% of the weight of the cement. Table 3 shows the various mix proportions used in this investigation.

**Table 3:** Mixture proportions used in the study.

Mix specification	Cement	M-sand	Coarse aggregate	Water	Conplast SP430	WPCB fibre	WPCB fibre (%)
	(kg/m^3^)						
Conventional concrete	412.5	780.39	991.68	164.96	4.125	0	0
AR30 1	412.5	780.39	991.68	164.96	4.125	4.13	1
AR30 2	412.5	780.39	991.68	164.96	4.125	8.25	2
AR30 3	412.5	780.39	991.68	164.96	4.125	12.38	3
AR30 4	412.5	780.39	991.68	164.96	4.125	16.5	4
AR30 5	412.5	780.39	991.68	164.96	4.125	20.63	5
AR40 1	412.5	780.39	991.68	164.96	4.125	4.13	1
AR40 2	412.5	780.39	991.68	164.96	4.125	8.25	2
AR40 3	412.5	780.39	991.68	164.96	4.125	12.38	3
AR40 4	412.5	780.39	991.68	164.96	4.125	16.5	4
AR40 5	412.5	780.39	991.68	164.96	4.125	20.63	5

The WPCB fibre-reinforced concrete design mix was prepared and mixed in a drum-type tilting concrete mixer. After preparing wet mixing of concrete, fresh properties, such as slump, are determined per Indian Standard IS 1199-1959^82^. The characteristics of hardened concrete were assessed by pouring it into a mould, finishing the sample surface and allowing it to set for 24 h. Then, the samples were taken out of the mould and cured in water for 7, 14 and 28 days, which is the necessary lifetime for performing mechanical properties. The concrete cube of 100 mm was subjected to Compressive Strength (CS) test, and a cylinder of 100 mm diameter and 200 mm height were subjected to a split Tensile Strength (TS) test following BIS 1199-1959 standard^82^ A prism to test Flexural Strength (FS) was cast for 100 mm × 100 mm × 500 mm according to IS 1199-1959^82^. The mechanical properties of PCB fiber-reinforced concrete were tested under loading conditions and limits. The testing was carried out in accordance with BIS 546-2018^83^ .

### RSM analysis

The RSM is an experimental outcomes analysis method. The R^2^, R^2^ Adjusted, and R^2^ Expected numerals were used to calculate the significance of the model level using the RSM method. The F-value computed in this method determines the impact of the variables on the quantified outcomes. The greater F-value of a parameter, the larger effect on the experiment. The P-value indicates the significance of the model's output. For statistical significance, a P-value of less than 0.05 is required for the model or set of parameters to be valid^84,85^. The RSM strategy was implemented for this research using a demo version of Design Expert 13 software.

## Experimental results and discussion

### WPCB fibre-reinforced fresh concrete properties

#### Workability

**Figure 5:**
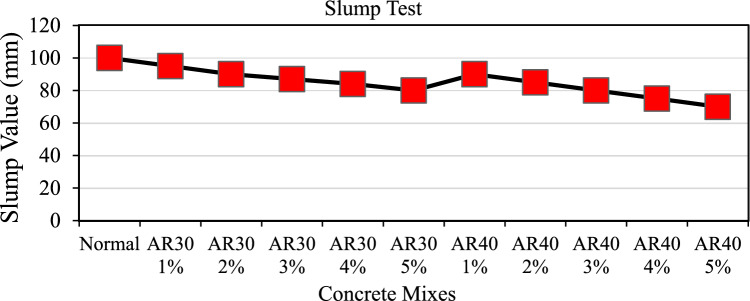
Slump values for different proportions of WPCB fibre-reinforced concrete mixes.

A slump test was used to determine the workability of freshly mixed concrete reinforced with WPCB fibres. Before casting the specimens, each mix was subjected to the slump test three times (in a single batch). Figure 5 depicts the slump test results. As the WPCB fibre content increases, the slump decreases almost in a straight line. As the aspect ratio (AR) increases, the slump value of the mix decreases. The slump value of WPCB fibre concrete dropped to 20 mm for adding 5% of AR30 WPCB fibres and 30 mm for adding 5% of AR40 WPCB fibres. An increase in the proportion of WPCB fibre in the mix reduced the workability, resulting in the mix's inconsistency. In addition, all mixes possessed a uniform distribution of randomly oriented discrete fibre without any evidence of a balling effect.

### Properties of WPCB fibre-reinforced hardened concrete

#### WPCB fibre-reinforced concrete compressive strength

**Figure 6:**
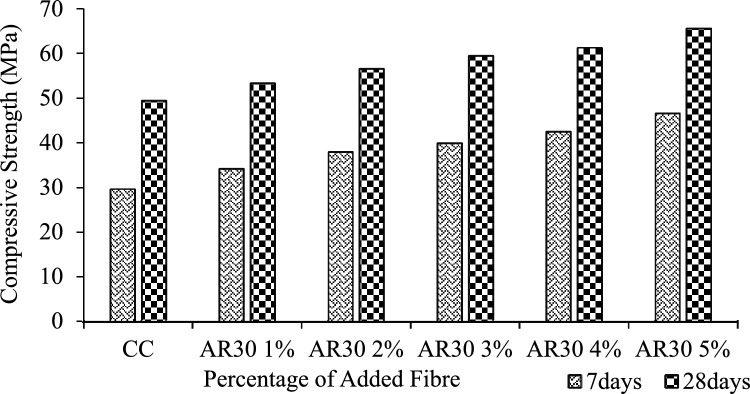
Compressive strength of AR 30 WPCB fibre-reinforced concrete mixes.

**Figure 7:**
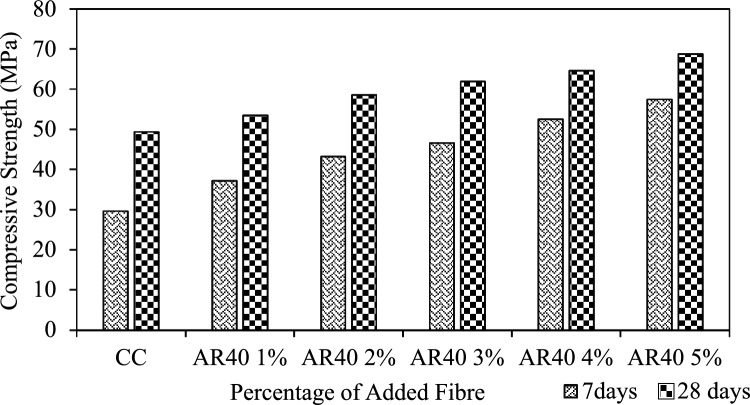
Compressive strength AR 40 WPCB fibre-reinforced concrete mixes.

The compression test results for 7-day and 28-day cured AR30 and AR40 WPCB fibre-reinforced concrete mixes are shown in Figs. 6 and 7, respectively. The design mix results demonstrated that the compressive strength increased steadily until 28 days for both AR30 and AR40 fibres. The compressive strength was 29.61 and 49.35 MPa at 7 and 28 days for the control concrete mix.

**Figure 8:**
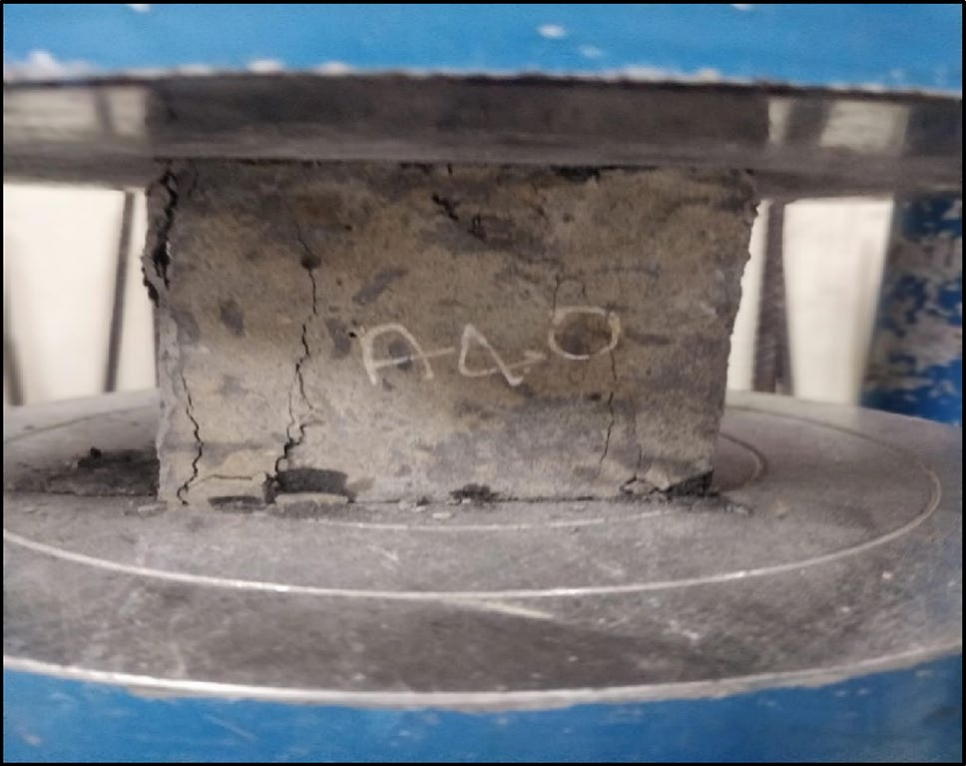
Failure of WPCB fibre-reinforced cube under compression.

For all the AR30 fibre mixes, the compressive strength at 28 days showed higher values than control concrete. The compressive strengths of all the AR30 WPCB fibre-reinforced concrete mixes are shown in Fig. 6. For the addition of 1%, 2%, and 3% of WPCB fibres, the compressive strength of the AR30 mix increased by 8.11%, 12.73% and 16.99%, respectively, compared to control concrete. Similarly, for the addition of 4% and 5% of WPCB fibres, compression strength increased by 19.43% and 24.71%, respectively. For all the five mixes prepared using AR40 fibres, the compressive strength of the 28-day cured WPCB fibre-reinforced concrete followed a similar trend as AR30 fibres. The compressive strength of AR 40 WPCB fibre-reinforced concretes are shown in Fig. 7. For 1%, 2% and 3% addition of WPCB fibre to the AR40 mix, the compressive strength increased by 8.31%, 18.64% and 25.39%, respectively, compared to conventional concrete. Similarly, a 4% and 5% addition of fibre increases the compressive strength by 30.86% and 39.37%, respectively. Adding WPCB fibre to the concrete progressively improved the compressive strength, as the increase in both aspect ratio and the percentage of WPCB fibre increases compressive strength. The increased aspect ratio from AR30 to AR40 of fibre results in a maximum increase in compressive strength for a 5% addition of WPCB fibre because of the availability of a more significant number of fibres in the mix^74^. Figure 8 shows the failure of WPCB fibre-reinforced cube under Compression. The increased compressive strength was because of the higher bonding between the WPCB fibre and concrete matrix due to increase in the quantity of WPCB fibres, However, a further increase in fibre percentage was causing a problem in achieving consistent mic proportion, as mentioned in workability section. This concrete did not exhibit brittle failure in spite of archive strength varying from 50 to 65Mpa. Additionally, this could be a cost-effective method for disposing of waste WPCBs.

#### WPCB fibre-reinforced concrete splitting tensile strength

**Figure 9:**
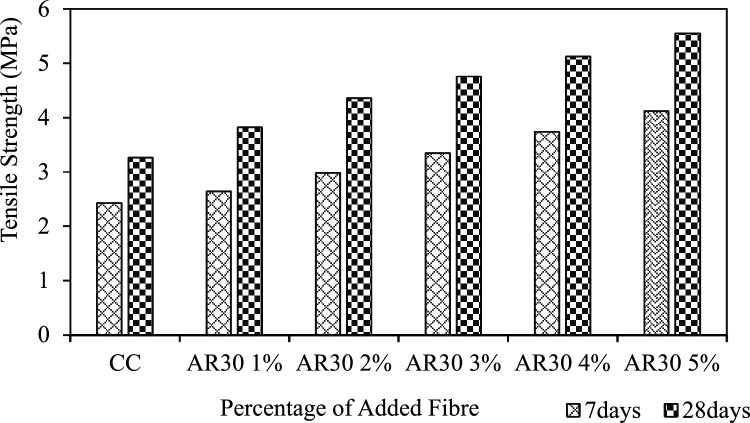
Tensile strength of AR30 WPCB fibre-reinforced concrete mixes.

**Figure 10:**
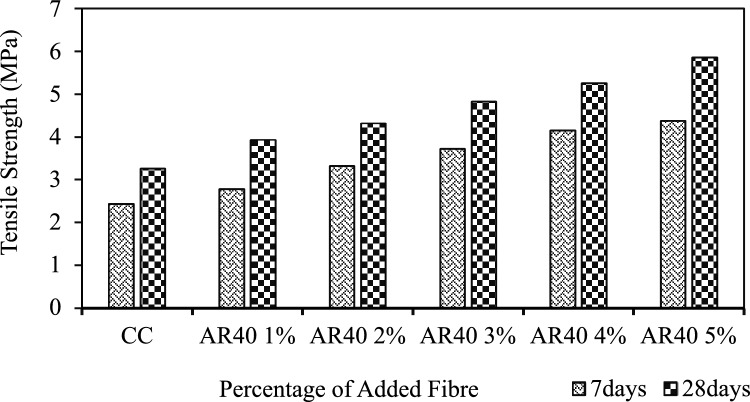
Tensile strength of AR40 WPCB fibre-reinforced concrete.

An earlier study also reported WPCB fibre-reinforced concrete's tensile strength^74^. The resistance of the concrete against its elongation is its tensile strength. The tensile strength of the AR30 and AR40 WPCB fibre-reinforced concrete are shown in Figs. 9 and 10. The tensile strength of the concrete after 28 days of curing, to which 1%, 2%, 3%, 4% and 5% of WPCB fibres are added, is higher than control concrete.

**Figure 11:**
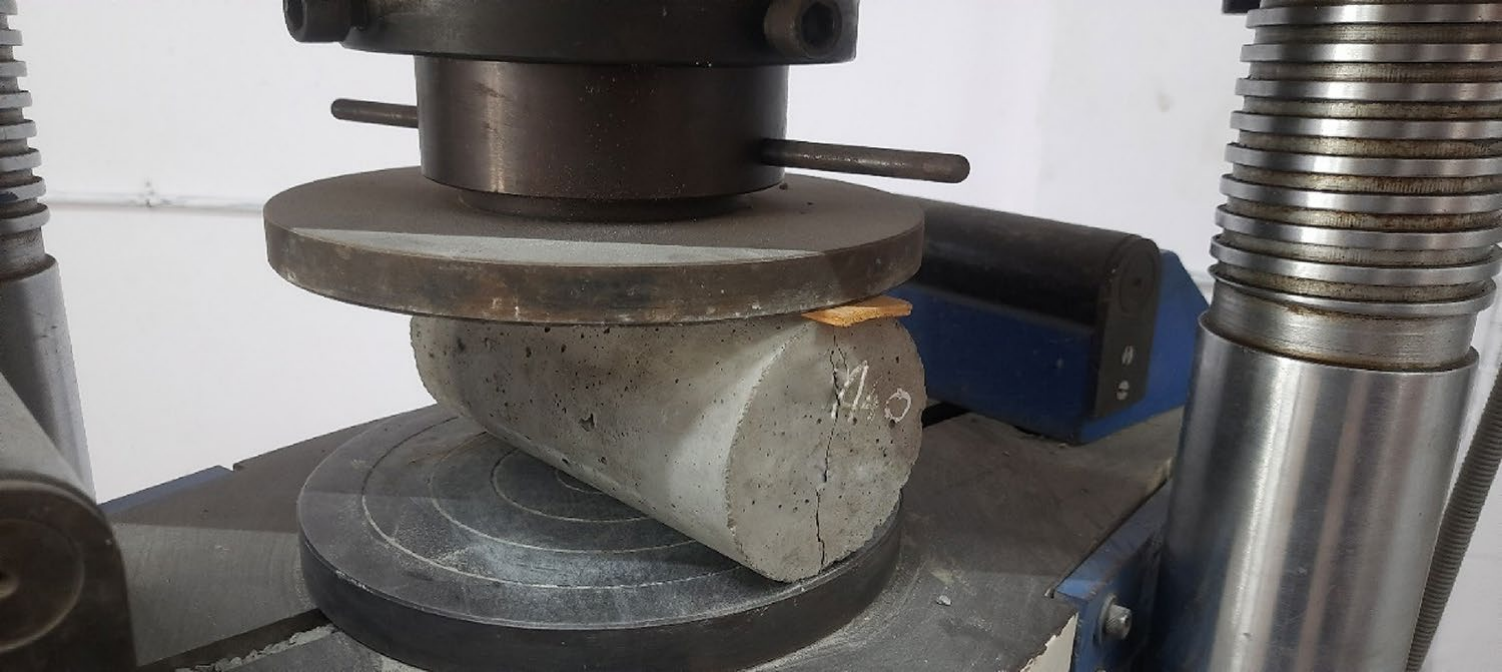
Failure of WPCB fibre-reinforced cylinder under split tension.

Adding 1%, 2%, 3%, 4%, and 5% of AR30 WPCB fibres to concrete increased the tensile strength of the resultant mixes by 17.18%, 33.74%, 46%, 57%, and 70.25%, respectively, when compared to control concrete. For example, from Fig. 9, it is seen that on adding 4% and 5% of WPCB fibres, the tensile strength of the mix was more than 50% higher than that of conventional concrete. Adding 1%, 2%, 3%, 4%, and 5% of AR40 WPCB fibres increased the tensile strength of the resultant mixes by 20.55%, 32.52%, 47.85%, 61.04%, and 79.75%, respectively, then the control concrete (Fig. 10). Adding WPCB fibres at 3%, 4% and 5% increased the tensile strength by 40% more than the control concrete. The concrete reinforced with 5% AR30 and AR40 WPCB fibres displayed the highest tensile strength because of the availability of more WPCB fibres. Figure 11 shows the failure of WPCB fibre-reinforced cylinder under Split tension. The better tensile strength in the case of a mix reinforced with AR40 WPCB fibre was due to an increase in the number of WPCB fibres compared to that in AR30 fibres. Under the ultimate load, the WPCB fibre-reinforced concrete did not experience brittle failure.

#### WPCB fibre-reinforced concrete flexural strength

**Figure 12:**
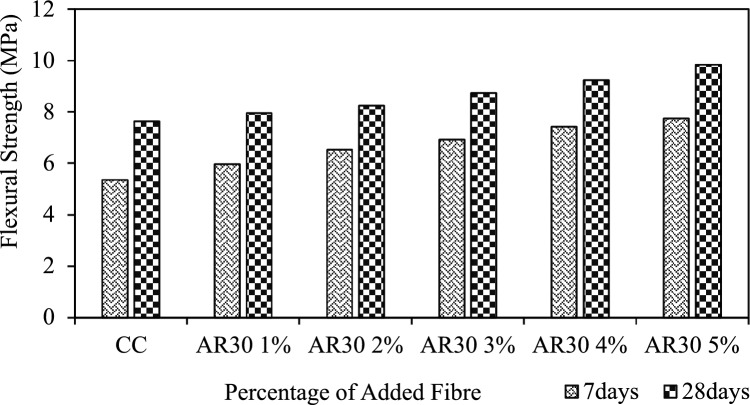
Flexural strength of AR 30 WPCB fibre-reinforced concrete mixes.

**Figure 13:**
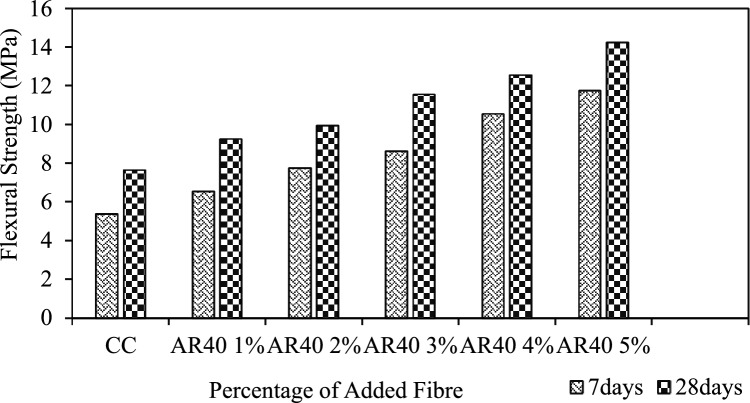
Flexural strength of AR 40 WPCB fibre-reinforced concrete mixes.

**Figure 14:**
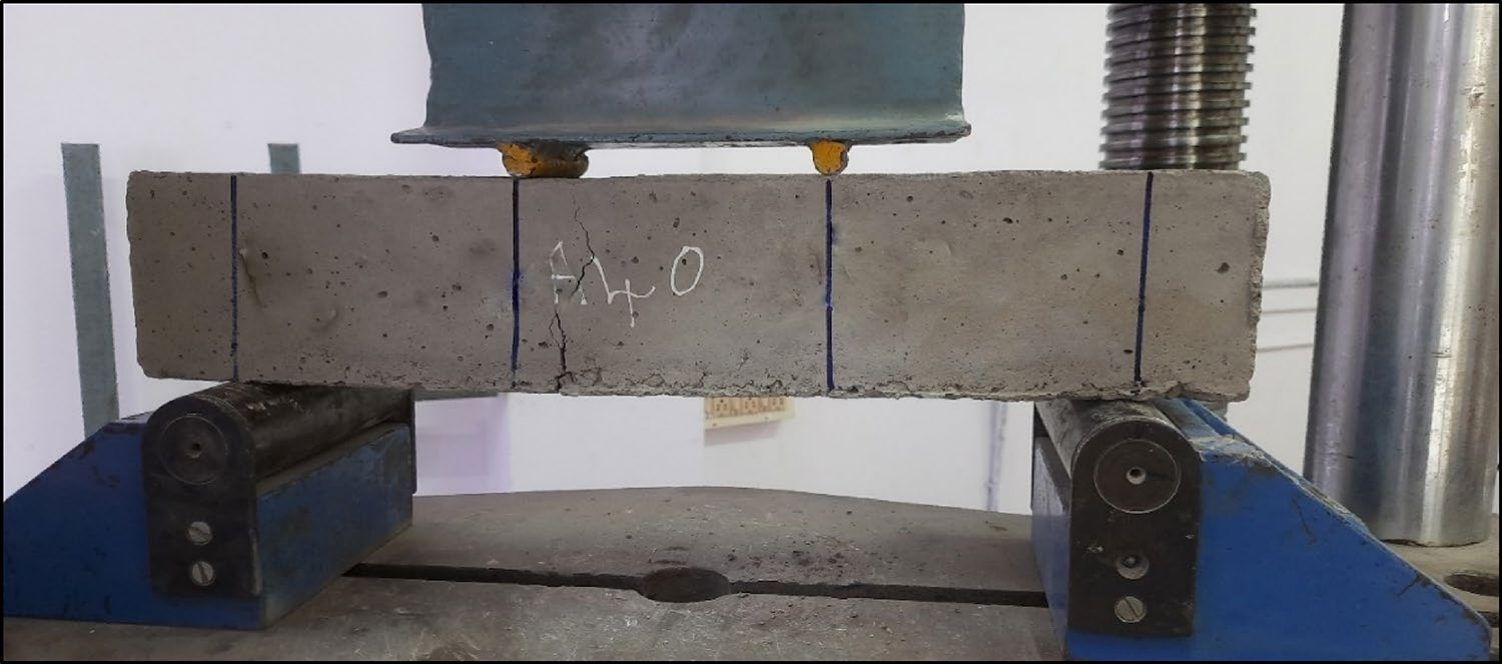
Failure of WPCB fibre-reinforced prism under flexure.

WPCB fibre improves the specimen's load-bearing capacity compared to the control specimen. This study demonstrated the similarity pattern between flexural strength and compressive strength. The flexural strength variations on adding 1%, 2%, 3%, 4% and 5% of AR30 WPCB fibre to the mix are shown in Fig. 12. Similarly, the flexural strength of the mix reinforced with AR40 WPCB fibre is depicted in Fig. 13. The mixes reinforced with AR30 and AR40 WPCB fibres show an increasing trend (Figs. 12 and 13) for flexural strength; this indicates that the flexural strength increases with the increase in the quantity of WPCB fibre. The highest flexural strength was attained for the 5% addition of WPCB fibre, as shown in Figs. 12 and 13, in which the flexural strength at 28 days for the mix reinforced with AR30 and AR40 fibres were 9.8 MPa and 14.25 MPa, respectively. Figure 14 shows the failure of WPCB fibre-reinforced Prism under Flexure. All five mixes reinforced with AR 40 fibres produced better flexural strength than those reinforced with AR30 fibres because of the availability of more WPCB fibres. Conversely, adding more than 5% of the fibre to the mix creates a balling effect of the WPCB fibre, reducing workability.

### Durability test

#### Acid test

**Figure 15:**
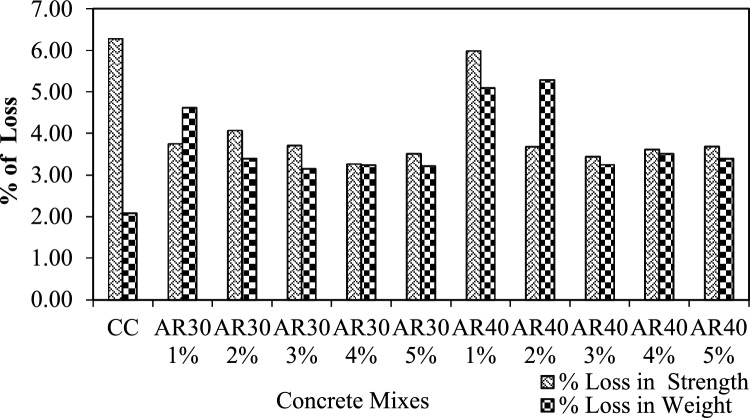
Acid test results of AR 30 and AR 40 WPCB fibre-reinforced concrete mixes.

Chemical attacks on concrete can cause physical effects such as increased porosity and permeability, reduced strength, cracking and spalling. In most cases, the chemical and physical deterioration processes work together simultaneously, and one process may occasionally contribute to accelerating the other processes. Therefore, an acid attack test was conducted on WPCB fibre-reinforced concrete to determine its chemical resistance. A 5% hydrochloric (HCl) acid was used for this experiment, and a 100 mm cube cured for 28 days was employed. Then the samples were cured in HCl for 30 days. After 15 days, the solution was changed to retain its concentration. After 30 days, specimens were collected from acid and air-dried. The samples were evaluated to determine the loss in weight (%) and then tested using a CTM. Figure 15 explains the loss in weight and compressive strength after exposure to the acidic environment for both AR30 and AR40 WPCB concrete-reinforced mixes. Test results indicate that mixes reinforced with 3%, 4% and 5% WPCB fibres show better performance in terms of a decline in strength and weight because of the availability of more fibres. The deterioration was mainly due to the bonding failure between the WPCB fibre and cement matrix in an acid environment. However, no significant strength loss was found in the WPCB fibre-reinforced concretes compared to control concrete.

#### Rapid chloride penetration test

A disc was cut from a 100 × 200 mm concrete cylinder with a thickness of 50 mm used for this experiment and cured for 30 days. The rapid chloride penetration test (RCPT) was performed following ASTM C1202-2019^86^. The RCPT was conducted by subjecting the specimen to a 60 V direct current for 6 h as per ASTM C1202-2019^86^. One reservoir has a solution of 3.0% NaCl, and the other has a solution of 0.3 M NaOH. Finally, a calculation is made of the total amount of charge passed. This test is widely reported in the literature and has been acknowledged as a standard^74,87^.

**Figure 16:**
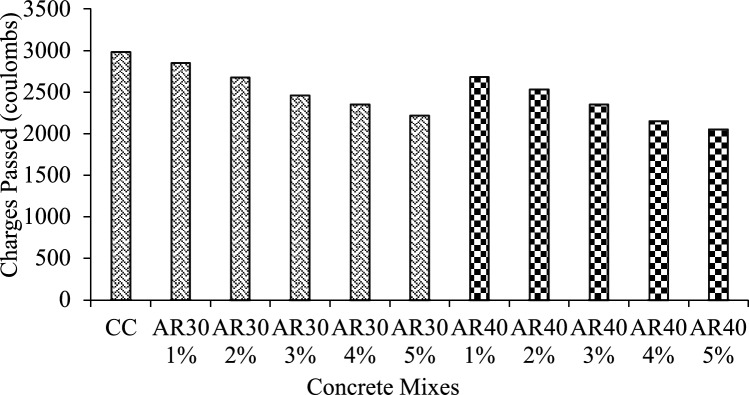
Rapid chloride penetration results of AR 30 and AR 40 WPCB reinforced concrete mixes.

Figure 16 depicts the chloride penetration values (in percentages) of AR30 and AR40 WPCB fibre-reinforced concretes. Chloride permeability is estimated to be between 2050 and 2981 coulombs. As per ASTM 1202-2019^86^, the distribution of chloride penetration was modest, so all WPCB fibre-reinforced concrete mixes achieved better resistance to chloride penetration. With the addition of WPCB fibres, the amount of charge being conducted through the concrete samples decreased for all five mixes reinforced with AR30 and AR40 WPCB fibres, indicating that the permeability of the concrete decreased. The chloride penetration abilities of concrete mixes with WPCB fibres are lower than control concrete. All WPCB fibre-reinforced concrete mixes have a moderate capacity for chloride diffusion. The addition of fibre does not affect the porosity of concrete. The AR40 WPCB concrete mixes show comparatively more significant performance characteristics. The reduction in RCPT value can be attributed to the reduction in porosity with reduced chloride diffusion.

#### Sorptivity

Sorptivity is the method for assessing the quantity of unsaturated flow of liquids into concrete on an unsaturated specimen or the capacity of a sample to absorb and transmit water through capillary action^88^. The sorptivity test gives valuable information about the presence of pore structures in concrete^89^. The laboratory test measures the water absorption rate by concrete samples according to ASTM C1585-2013^90^. The only requirements for this test are a pan of water, a stopwatch and a ruler. Then a specimen was cut from a 100 mm diameter cylinder with a thickness of 50 mm and cured for 28 days employed in the test. The sides of the specimen were insulated with electrical tape. The mass is initially recorded, and the specimen is immersed in water at a depth of 5–10 mm for 0.6 s. Samples were checked at 1, 2, 3, 4, 6, 9, 12, 16, 20, 25, 30 and 45 min and 1-h intervals. Then, the sample was taken out of the water at specified intervals.

**Figure 17:**
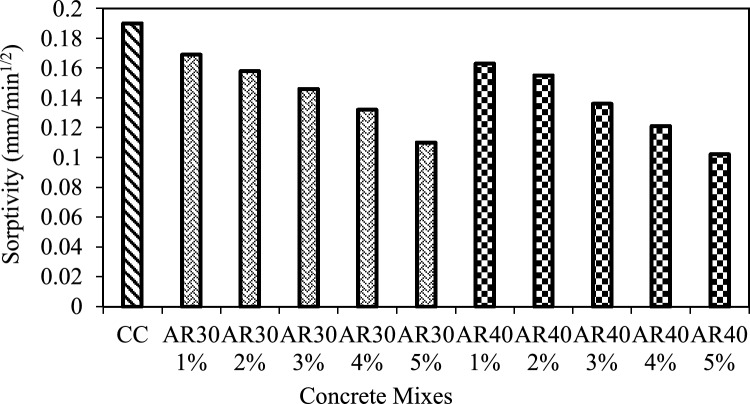
Sorptivity test results of AR30 and AR40 WPCB fibre-reinforced concrete mixes.

The square root of the time elapsed versus the mass obtained per unit area relative to water density was plotted. The slope for the best-fitting line (after eliminating the origin) was interpreted as sorptivity. For analysing the sorptivity index (m^3^ × 10^–7^/ min^(1/2)^), the graph is plotted between the square root of time and flow quantity. Figure 17 shows that all the AR30 and AR40 WPCB fibre-reinforced concrete mixes exhibit good sorption. The water absorption rate is due to the proper distributions of WPCB fibre that clog the concrete pores, preventing water absorption. As the proportion of WPCB fibre increases, the sorptivity index value decreases for both AR30, and AR40 WPCB fibre-reinforced concretes. Lower sorptivity values indicate that the concrete is more resistant to water absorption.

## RSM results of WPCB fibre-reinforced concrete

This investigation uses a response surface model (RSM), which is trained to predict the properties like tensile strength, compressive strength and flexural strength of WPCB fibre-reinforced concrete. The accuracy of each of their predictions is measured and compared.

The functional relationships between the response of interest $\left(y\right)$ and input variables $\left(x\right)$ Were modelled using statistical methods of RSM^91,92^. The RSM polynomial model as a function of the input variable $\left(x\right)$, output variable $\left(y\right)$, random experimental error with a zero mean (ε) and a vector of unknown constant coefficients (β) is given in Eq. (1)^93^.

1 $$y={\beta }_{0}+\sum_{i=1}^{k} {\beta }_{i}{x}_{i}+\sum \sum_{i<j} {\beta }_{ij}{x}_{i}{x}_{j}+\sum_{i=1}^{k} {\beta }_{i}{x}_{i}^{2}+\varepsilon$$

In this study, eight performance indicators were chosen to evaluate prediction results obtained from RSM. The metrics consist of the coefficient of determination (${R}^{2}$), the adjusted ${R}^{2}$, the Root-Mean-Square Error (RMSE), the Mean Absolute Error (MAE), the Mean Relative Error (MRE), the Mean Absolute Percentage Error (MAPE) and the relative Root-Mean-Square Error (rRMSE).

Table 4 displays the assessment metrics based on the prediction algorithms, in response surface model, and the analysis of variance results for the 28^th^-day mechanical properties of concrete cube cylinders of 100 mm diameter. At the 5% significance level, the variance analysis offers the sum of squares,* p*-value, *F*-value, mean squares and Degree of Freedom (DoF). It was found that ${R}^{2}$ value was greater than 0.98 for all the tested mechanical properties of WPCB fibre-reinforced concrete. To prevent the *R*^2^ from being artificially inflated by introducing new variables, an adjustment or modification known as the adjusted *R*^2^ has been developed (this means that the adjusted *R*^2^ value is higher because the presence of only critical variables affects the physical interpretation of the response). It is desirable if the modified ${R}^{2}$ is within 0.2 of the ${R}^{2}$. The assessment served as the foundation for the investigation. Moreover, the model's *p*-value of less than 0.5 implies that it is statistically significant^94^.

**Table 4:** Statistical comparison of the RSM model for WPCB fibre-reinforced concrete.

Statistics	Compressive strength		Tensile strength		Flexural strength	
Metrics	AR30	AR40	AR30	AR40	AR30	AR40
${R}^{2}$	0.982	0.9871	0.9802	0.9921	0.9844	0.9883
Adjust ${R}^{2}$	0.976	0.9828	0.9869	0.9895	0.9791	0.9844
RMSE	1.196	1.165	1.245	1.365	1.354	1.485
rRMSE (%)	24.21	23.25	26.21	27.25	28.21	30.25
MAPE (%)	12.36	13.36	13.36	15.36	16.36	18.36
MRE	0.042	0.038	0.039	0.042	0.041	0.042
MAE	0.114	0.11	0.113	0.11	0.115	0.274
MSE	0.015	0.012	0.017	0.015	0.019	0.082

**Figure 18:**
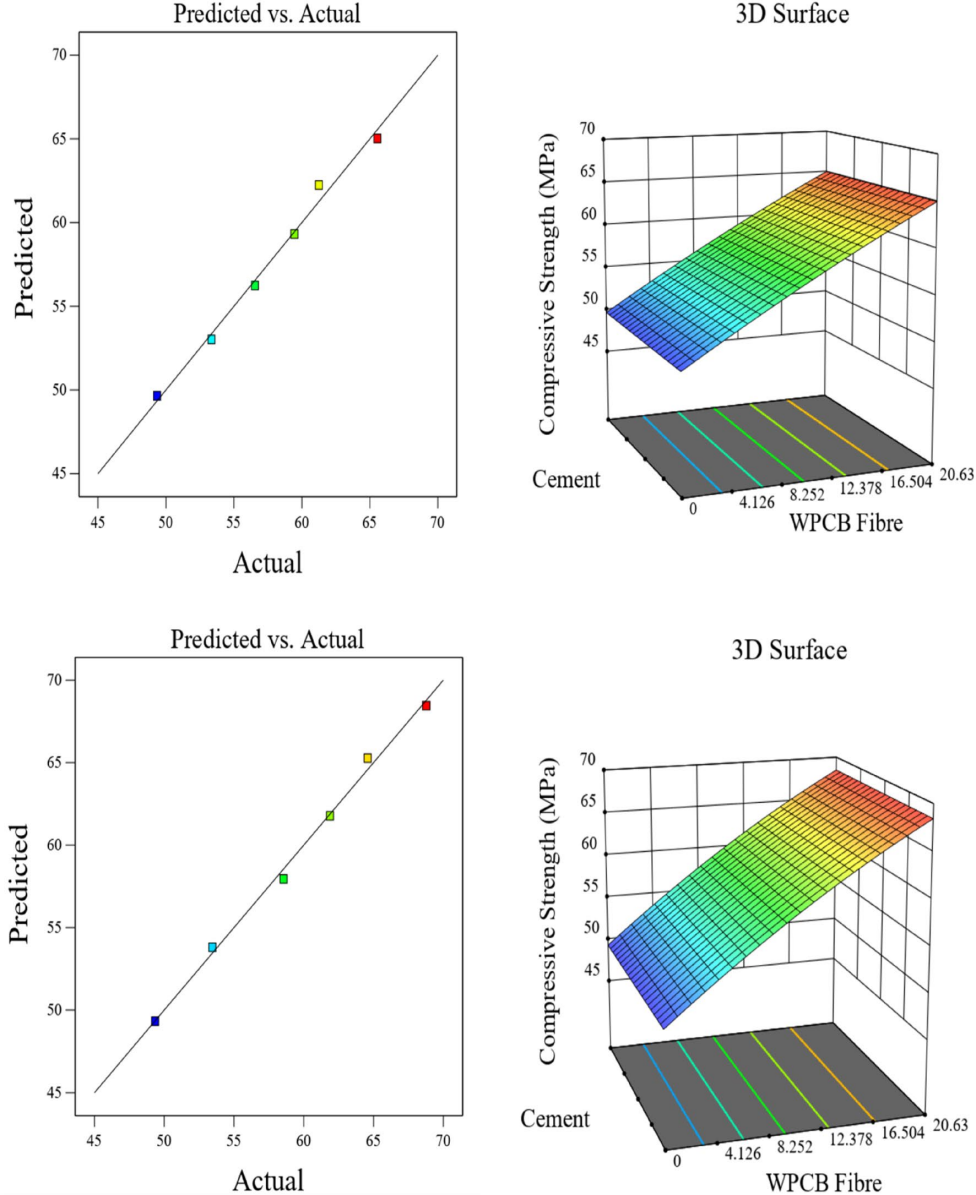
Actual versus prediction graphs and surface plots for compressive strength of WPCB fibre of AR30 and AR40.

**Figure 19:**
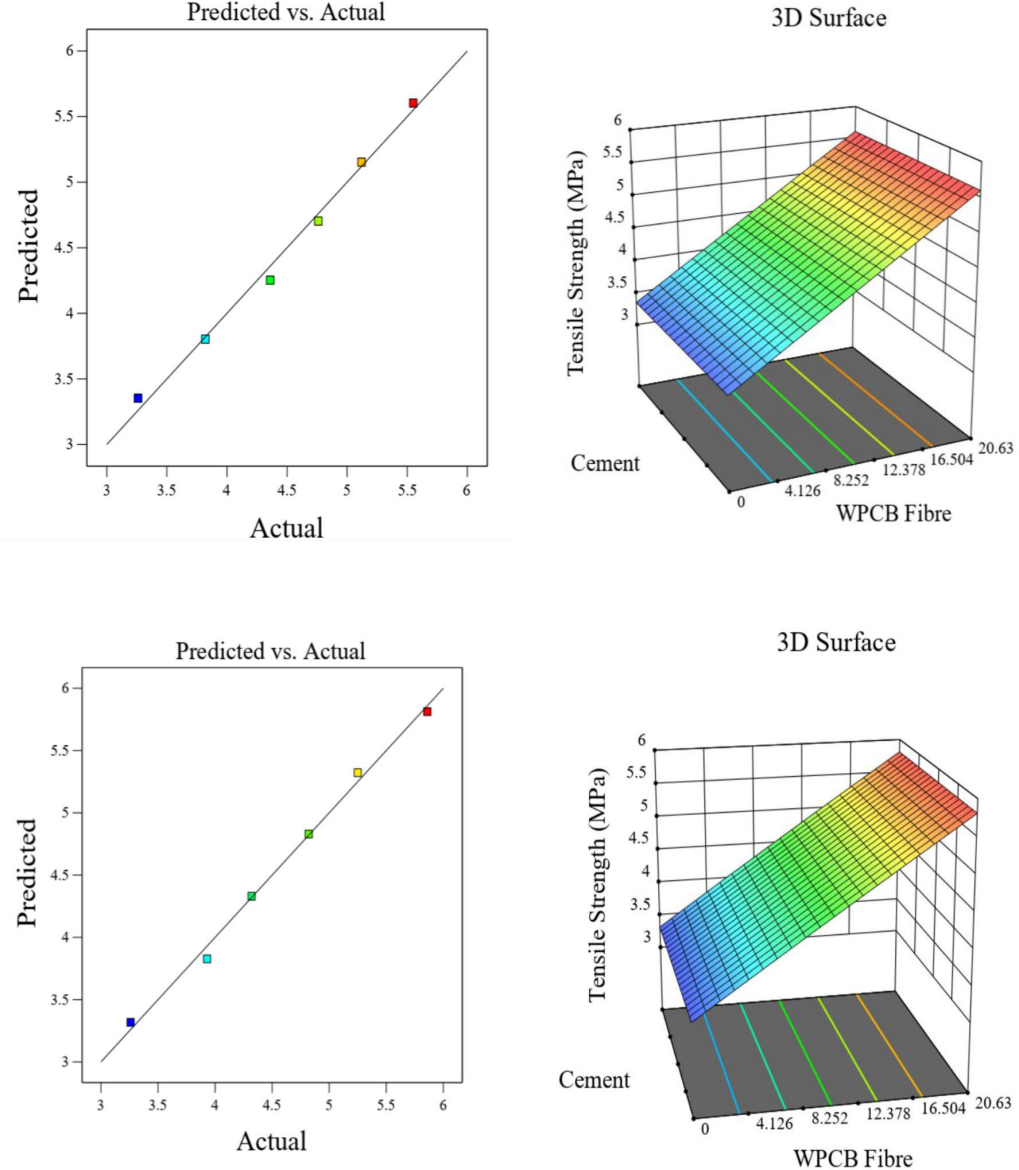
Actual versus prediction graphs and surface plots for tensile strength of WPCB fibre of AR30 and AR40.

**Figure 20:**
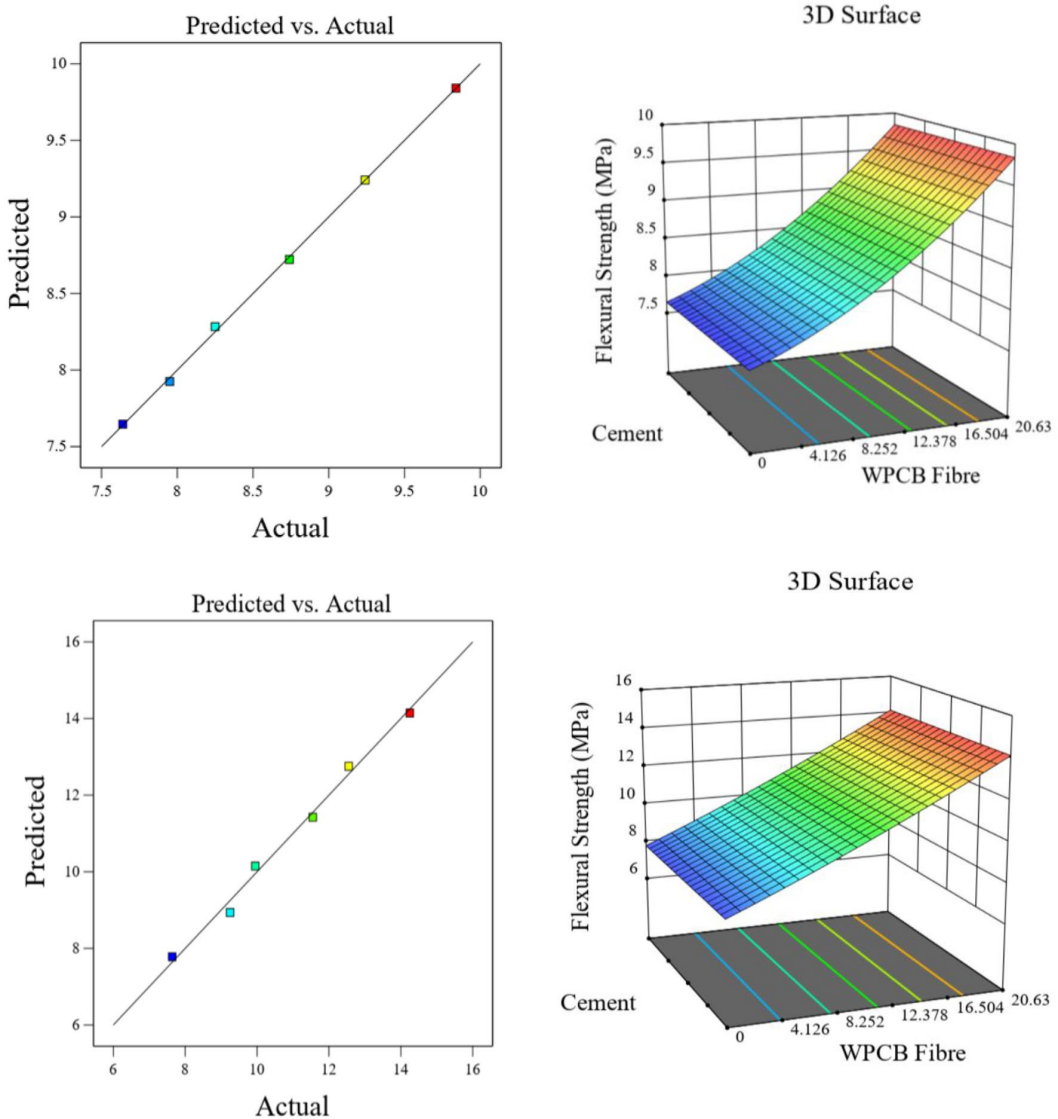
Actual versus prediction graphs and surface plots for flexural strength of WPCB fibre of AR30 and AR40.

Figures 18, 19 and 20 compare the predicted and actual values and the 3D surface plots of the three mechanical properties (compression, Tension and flexure) of the WPCB fibre-reinforced concrete. It was found that the predicted values are close to the fit line (diagonal) for all the values of the three mechanical properties of the WPCB fibre-reinforced concrete. The even spread of values below and above the fit line (diagonal) indicates that the RSM model correctly predicted the outcome. Furthermore, it that the RSM model has no under-prediction or over-prediction bias.

The 3D surface plots of the strength parameters also clearly show that the increase in fibre content tends to increase the mechanical properties of WPCB fibre-reinforced concrete. The RSM model's main weakness is that it is aliased, which means estimating a factor's influence affects the element.

The closeness of the predicted and actual responses is demonstrated by the actual and predicted response values. The residual plots did not show any significant deviation from normality; the plots clearly show that the chosen model could predict properties and connections between the materials used.

## Conclusions

WPCBs converted as fibre is an alternative to conventional fibres, reducing the environmental hazards. WPCBs fibre-reinforced concrete mechanical properties were thoroughly investigated. The contribution of WPCB fibre to fresh and hardened concrete properties and durability properties was determined experimentally, and the experimental results were validated using a statistical model developed with RSM. The conclusions are drawn from this study:

1. Mechanical properties such as compressive strength, tensile strength and flexural strength were assessed for WPCB fibre-reinforced concrete in various proportions (1%, 2%, 3%, 4%, and 5%) for two different aspect ratios of AR30 and AR40 of WPCB fibres. Compared to control concrete, the compressive strength of WPCB fibre-reinforced concrete increased by 32.8% for AR30 and 40.8% for AR40 of WPCB fibres due to more fibres in the composite.
2. In AR30, tensile strength is 70% and 80.1% for AR40 of WPCB-reinforced concrete compared to conventional concrete. The trend is nearly identical to compressive strength test results obtained for tensile strength and flexural, which could be strongly linked with increased WPCs fibres in the AR40 concrete mixes.
3. Acidic environment's bond breakdown between WPCB fibres and the cement matrix, it is revealed that the AR30 concrete mixes were found to be more durable than AR40 concrete mixes due to the presence of a higher bonding area which requires a relatively more period for deterioration. The RCPT test confirmed that WPCB fibre conserved porosity, and the AR40 mixes performed much better, demonstrating that the WPCB fibre mix can produce high-performance concrete with low porosity and higher chloride resistance. The sorptivity index value drops for AR30 and AR40 mixes as the percentage of WPCB fibre increases. However, AR40 performed much better than AR30. Lower sorptivity numbers mean that the concrete is less likely to absorb water.
4. The RSM accurately validated the mechanical properties of the WPCB fibre-reinforced concrete (${R}^{2}$ ≥ 0.98). Furthermore, the 3D surface plot also reveals that increased fibre content directly increases the mechanical properties like compressive strength, tensile strength and flexural strength of the WPCB fibre-reinforced concrete.
5. Adding WPCB fibre improves the properties of fresh and hardened concrete significantly. The solid bonding strength between the fibre and matrix phase was the reason for the strength increase of WPCBs fibre-reinforced concrete.

In conclusion, this research suggests that the utilization of hazardous WPCB as construction material with long term durability, could be effective waste management and reduce negative environmental impacts.

## Limitation and scope for further work

- Beyond 5% addition of WPCB fibre is cause the balling effect, which directly affects the workability nature of the concrete mix, which reduces the mechanical properties of the designed grade of concrete.
- The Balling effect may be avoided by decreasing the aspect ratios of the WPCB fibre, which increase the utilization of more WPCB fibre in concrete that may result in resisting the crack propagation in the concrete member.
- A limitation in the mechanical process has forced the investigators to study the properties of concrete by incorporating PCB Fiber with a width of 1 mm to 1.5 mm. a refinement in the process a manufacturing PCB fibre with a width of 0.1 to 0.5 mm can yield better results breeding the micro and macro cracks.
- The work was focused only on WPCB fiber addition in standard cement concrete; however, there were limitations in cutting the fiber to lower width/ thickness, making it challenging to maintain AR80 followed in conventional fiber-reinforced concrete.
- It is evident that WPCB might contains heavy metals in spite of removal external metal components and hence a comprehensive life cycle assessment would be helpful in assign the advantage and disadvantage in utilizing the same material in concrete as obsessed to traditional disposal methods.
- Fire resistance characteristics of reinforced WPCB concrete would prove beneficial as PCB contains epoxy materials

## Data Availability

The datasets generated and analyzed during the current study are available from the corresponding author on reasonable request.
